# Pink shrimp *Farfantepenaeus duorarum* spatiotemporal abundance trends along an urban, subtropical shoreline slated for restoration

**DOI:** 10.1371/journal.pone.0198539

**Published:** 2018-11-07

**Authors:** Ian C. Zink, Joan A. Browder, Diego Lirman, Joseph E. Serafy

**Affiliations:** 1 Cooperative Institute for Marine and Atmospheric Science, Rosenstiel School of Marine and Atmospheric Science, University of Miami, Miami, FL, United States of America; 2 Protected Resources and Biodiversity Division, Southeast Fisheries Science Center, National Marine Fisheries Service, National Oceanic and Atmospheric Administration, Miami, FL, United States of America; 3 Marine Biology and Ecology Department, Rosenstiel School of Marine and Atmospheric Science, University of Miami, Miami, FL, United States of America; Fred Hutchinson Cancer Research Center, UNITED STATES

## Abstract

The Biscayne Bay Coastal Wetlands (BBCW) project of the Comprehensive Everglades Restoration Plan (CERP) aims to reduce point-source freshwater discharges and spread freshwater flow along the mainland shoreline of southern Biscayne Bay. These actions will be taken to approximate conditions in the coastal wetlands and bay that existed prior to construction of canals and water control structures. An increase in pink shrimp (*Farfantepenaeus duorarum*) density to ≥ 2 individuals m^-2^ during the wet season (i.e., August-October) along the mainland shoreline was previously proposed as an indication of BBCW success. This study examined pre-BBCW baseline densities and compared them with the proposed target. Densities were monitored by seasonal (wet, dry) throw-trapping (1 m^2^ replicated in triplicate) at 47 sites along ~22 km of the southwestern Biscayne Bay coastline over 10 years (2007–2016). Densities varied across years and were most often higher in dry seasons. Quantile regression revealed density limitation by four habitat attributes: water temperature (°C), depth (m), salinity (ppt), and submerged aquatic vegetation (SAV: % cover). Procrustean analyses that tested for concordance between the spatial and temporal distributions of shrimp densities and habitat metrics found that water temperature, water depth, and salinity explained ~ 28%, 28%, and 22% of density variability, respectively. No significant relationship with SAV was observed. Hierarchical clustering was used to identify spatially and temporally similar groupings of pink shrimp densities by sites or season-years. Significant groupings were then investigated with respect to potentially limiting habitat attributes. Six site and four year-season clusters were identified. Although habitat attributes significantly differed among spatial clusters, within-cluster median pink shrimp densities did not correlate with within-cluster minima, maxima, medians, or standard deviations of habitat attributes. Overall, pink shrimp density ($\bar{X}$ = 0.86, SD = 1.32 shrimp m^-2^) was significantly lower (t_(α = 0.10,2),939_ = -26.53, P <0.0001) than the 2 shrimp m^-2^ CERP Interim Goal target. Pink shrimp density corresponded significantly with salinity and appeared limited to density < 2 shrimp m-^2^ by salinity < ~18 ppt. Salinity is an environmental attribute that will be directly influenced by CERP implementation.

## Introduction

Biscayne Bay is a coastal lagoon located adjacent to the city of Miami, Florida, USA. Its watershed was heavily modified during the 20^th^ century and is currently highly managed to prevent urban, suburban, and agricultural flooding while also meeting agricultural, commercial, and residential freshwater demands. The Comprehensive Everglades Restoration Plan (CERP) seeks to restore the quality, quantity, timing, and distribution of freshwater deliveries to southern Florida nearshore areas [1], including Biscayne Bay. The Biscayne Bay Coastal Wetlands (BBCW) project, a CERP component, is designed to restore a more natural hydrology and salinity regime along the bay’s southwestern shoreline [2,3]. Three actions are needed to make this improvement: (1) increasing the total volume of freshwater deliveries; (2) diverting part of point-source freshwater discharge (i.e., canal discharge) into coastal wetlands to enter the bay as overland sheet flow; and (3) altering the present timing of deliveries by extending discharges through the wet season (May-October) and into the dry season (November-April) [3,4]. The REstoration COordination and VERification (RECOVER) team established Interim Goals (IGs) to link ecological indicator metrics to CERP activities and thus evaluate restoration performance and realization of post-implementation ecological benefits at 5-yr intervals [1].

The pink shrimp *Farfantepenaeus duorarum* is one of many ecological indicators selected to assess ecological impacts of CERP implementation [1,5]. Pink shrimp was selected to assess effects of salinity change because previous work suggested linkage to salinity [1,5]. As reviewed by Zink et al. [6], higher pink shrimp productivity can be expected at salinities within polyhaline (18–30 ppt: [7]) and euhaline (30–40 ppt) ranges. Expansion of southwestern Biscayne Bay estuarine habitat was expected to benefit pink shrimp residing there [8]. Increased pink shrimp abundance might result indirectly from increased seagrass cover and spatial extent [5,8] that might occur from reduction in stress from extreme salinity fluctuations. Higher abundance of pink shrimp has been reported in areas exhibiting higher and more stable salinity [8–10], which also exhibit more continuous seagrass cover [11]. Areal expansion of shoal grass (*Halodule wrightii*) cover could further amplify pink shrimp abundance due to an apparent affinity for this seagrass species [12]. The stated pink shrimp IG for southwestern Biscayne Bay is “2 shrimp m^-2^ in nearshore optimal habitat (i.e., seagrasses)” during August-October peak abundance periods [1]. This IG was based upon a peak density of ~1.8 shrimp m^-2^ observed in September during a 2 yr (2003–2004) pilot study [8].

Historically, most freshwater delivery to Biscayne Bay was through transverse glades, broad natural channels through the Miami Coastal Ridge that allowed Everglades Basin surface water drainage [13,14] and groundwater seepage [15–20]. These natural drainage features fed fresh water from the Everglades through the coastal ridge via transverse glades into creek networks that spread surface water flows along the bay’s shoreline. Canalization converted the freshwater delivery system to one dominated by pulsed point-source (i.e., canal mouth) discharges that altered benthic submerged aquatic vegetation (SAV), infaunal, epifaunal, and nekton communities [11,21–26] and lowered the water table, reducing groundwater seepage [19,20].

**Fig 1:**
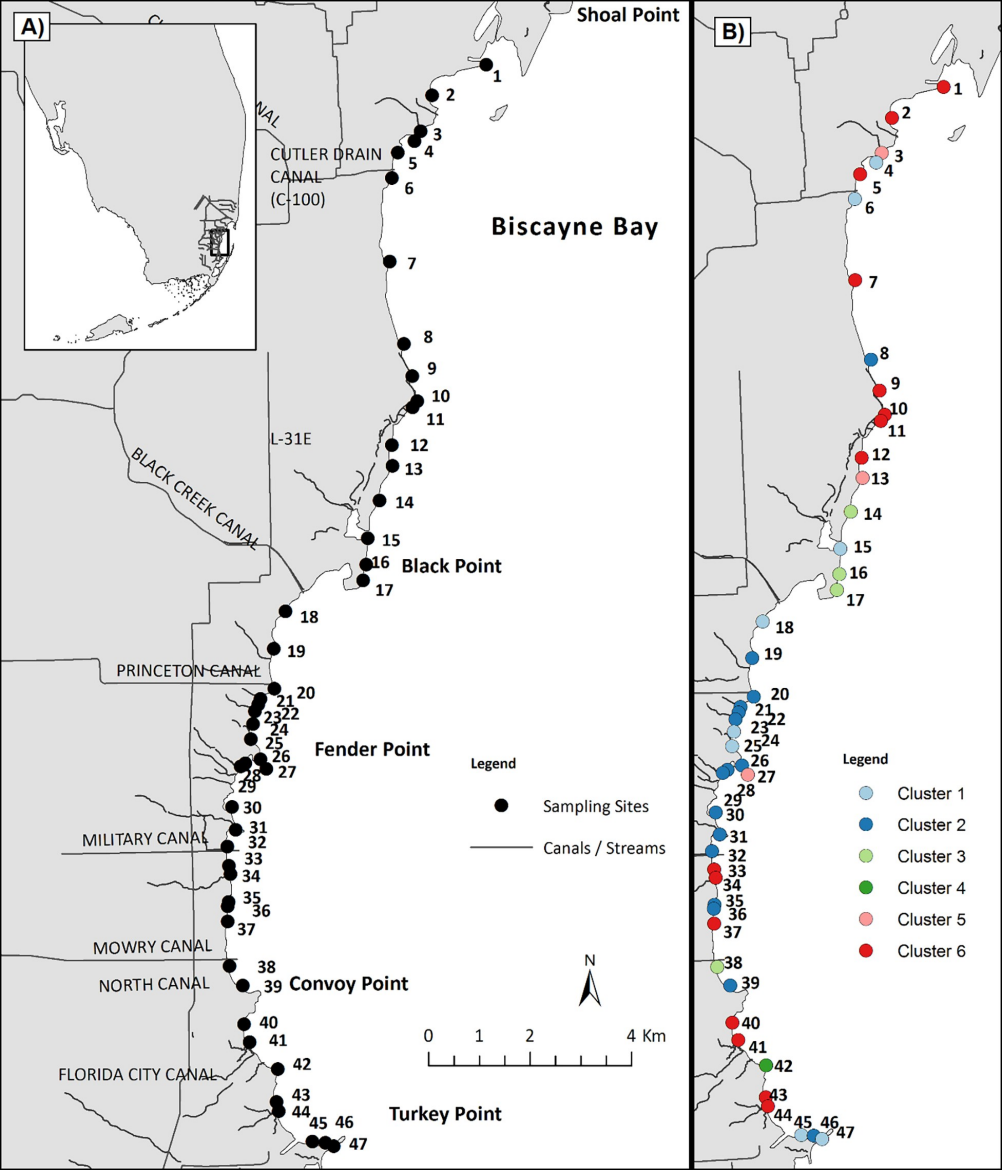
Map of study area, including referenced geographical features, and location of survey sites. The second panel depicts the same sites color-coded to match significant site clusters (Figs 2B and 4). Cluster 6 (red) exhibited similar spatial density patterns that were, on average, significantly higher than all other cluster groups. Conversely, Cluster 1 (light blue) exhibited significant, consistently lower densities than all other groups except for Cluster 4 (dark green). Clusters 2–5 exhibited intermediate average densities (Fig 4).

BBCW salinity goals for southwestern Biscayne Bay (Shoal Point to Turkey Point: Fig 1) prescribe oligohaline (0.5–5 ppt) and mesohaline (5–18 ppt) regimes at the shoreline, trending towards 20 ppt (polyhaline, 18–30 ppt) 500 m from the coast [4]. These conditions are anticipated to enrich estuarine faunal assemblages as well as increase estuarine species distributions and abundances [4,27,28]. Expansion of continuous submerged aquatic vegetation (SAV) habitats dominated by *Halodule wrightii*, a species commonly associated with low and variable salinity, is foreseen [11,24–26,29,30]. BBCW implementation goals for benthic habitat include increased spatial extent of nearshore seagrass beds, especially seaward expansion of *H*. *wrightii* [30]. Increased overlap of optimal salinity conditions with preferred benthic SAV habitats would provide synergistic benefits to estuarine fauna such as pink shrimp [4,5,27,31].

The purpose of this study was to investigate spatiotemporal trends in pink shrimp density along the southwestern Biscayne Bay shoreline. We investigated the plausibility of the post-CERP establishment of ≥ 2 shrimp m^-2^ IG. Further, we addressed presumptions that (1) pink shrimp peak abundance occurs during the wet season and (2) nearshore mesohaline salinity goals would yield increased pink shrimp abundance in the nearshore zone. Pink shrimp density relationships to species-specific and total SAV % cover and canopy height were also investigated. Our focus was on evaluating temporal (i.e., seasonal and inter-annual) and spatial (i.e., among sites) pink shrimp density trends relative to habitat attributes. This was achieved by (1) using quantile regression to identify habitat attributes that potentially limit pink shrimp density, (2) organizing pink shrimp density and habitat observations via heatmaps to visually assess spatiotemporal variability and trends, (3) using Procrustean analysis to measure concordance between shrimp density and habitat attribute matrices, (4) employing hierarchical clustering analysis to identify spatiotemporal density clusters, and (5) investigating distributional aspects (median, minimum, maximum, and standard deviation) of habitat attribute values (temperature, salinity, water depth, and SAV % cover) within density clusters to link density patterns to the environment. These analyses employ data from wet and dry seasons of 10 years of epifaunal community sampling at 47 sites within 50 m of shore spanning ~22 km of shoreline.

## Materials and methods

### Study area

Biscayne Bay is a large (1,110 km^2^), shallow (depths generally < 3 m), subtropical lagoon system extending approximately 56 km north to south along the southeast coast of Florida, USA (Fig 1). Where coastal urban development is low, the bay’s mainland shoreline consists of mangrove-seagrass ecotone punctuated by natural tidal creeks, freshwater canals, and other channels [32]. Overland freshwater discharges and groundwater seepage create a salinity gradient across the bay perpendicular to the shoreline with three salinity zones: (1) western nearshore areas usually affording the lowest salinities; (2) the bay central axis marked by near oceanic salinities; and (3) oceanic salinities near the eastern passes through barrier islands to the open ocean [24,29,33]. Tidal ranges are generally 0.5 to 1 m [34,35].

### Field surveys

Epifaunal communities and SAV habitats were surveyed seasonally at fixed sampling sites (n = 47) along the southwestern Biscayne Bay nearshore zone (0–50 m) from Shoal Point to Turkey Point (Fig 1). Surveys were conducted in public waters under authority of Biscayne National Park (Study #: BISC 06016, Permit #: BISC-2017-SCI0022). Surveys were conducted in dry (January-March sampling) and wet (July-September sampling) seasons that characterize south Florida’s climate. The primary sampling unit was the 20 m buffer around GPS coordinates that identified permanent sampling sites. Sites were located in the shallow, open water along the western shoreline mangrove-seagrass ecotone, an area likely to be directly affected by CERP implementation. During each survey, the 47 fixed sampling sites were visited within 3 hr of high tide over 5 to 7 days within a 2 week period. Water quality and habitat parameters, including water temperature (°C), salinity (ppt), pH, dissolved oxygen saturation (%), dissolved oxygen concentration (mg L^-1^), water depth (m), and sediment depth (m), were recorded at each site. This study analyzed farfantepenaid and associated chemical and physical sampling data collected from 2007 through 2016.

Benthic habitats were assessed for species-specific SAV % cover by visual assessment of 10 replicate 0.5 m^2^ quadrats per site [24,25]. In addition, canopy height (maximum seagrass blade length) was measured to provide a topography metric. Species-specific and total SAV % cover data following the methods of Lirman et al. [25] were obtained for the period 2008 to 2016.

Epifaunal communities were sub-sampled at each site (n = 3) using an open-ended, rigid-sided aluminum box (i.e., throw-trap) measuring 45 cm tall by 1 m^2^ [36,37]. Two 3-mm stretch-mesh cover nets affixed to opposite sides of the throw-trap upper surface prevented epifauna escape during deployment in water deeper than 45 cm. Once deployed, the throw-trap was cleared of trapped epifauna by sweeping (n = 4) its interior from alternating directions with a metal-framed seine fitted with 3 mm stretch-mesh, while gently tapping the substrate with the seine frame. Organisms collected from each sub-sample throw-trap deployment were bagged and numbered separately for storing and processing. Samples were frozen during storage until processing. No protected species were sampled.

### Epifauna identification and measurement

Taxonomic identifications and size measurements were conducted in the laboratory. Organisms collected from each replicate throw-trap deployment at a site were maintained and processed independently. Where possible, carapace length (CL, mm) and total length (TL, mm) were recorded for each farfantepenaeid shrimp. Shrimps >8.0 mm CL were identified to species primarily using petasma and thelycum (i.e., sexual) morphology, although other characteristics were also used [38–41]. Shrimps <8.0 mm CL were identified to genus due to low degree of sexual morphological development [39].

### Statistical analysis

All statistical analyses were performed using the R statistical package (The R Foundation, https://www.r-project.org/). Statistical analyses were performed with a Type 1 error criterion of α = 0.10 to reduce potential Type 2 errors. Shrimp density (#/ m^2^) at each site was calculated as the sum of observed shrimps from the triplicate throw-trap sub-samples divided by 3 m^2^. Density data were natural logarithm (x + 1) transformed before analysis to reduce influence of outliers.

#### Potential habitat limitations on pink shrimp density

As a statistical interpretation of the ecological concept of Leibig’s Law of the Minimum [42,43], quantile regression (QR) has been applied to identify species distribution or abundance limitation by specific habitat attributes by focusing specifically on the upper bound of the abundance vs. habitat attribute relationship [44–47]. Pink shrimp density was first plotted against individual habitat factors to graphically assess potential limiting factors. QRs (function ‘rq’ of package ‘quantreg’) fit to the 0.5 and 0.9 density percentiles were used to statistically identify a subset of habitat attributes that suggested limitation at the median and upper edge of the density distribution. Analyses initially considered water temperature (°C), salinity (ppt), pH, dissolved oxygen saturation (%), dissolved oxygen concentration (mg/L), water depth (m), sediment depth (m), and the following SAV metrics: *Thalassia testudinum* % cover, *H*. *wrightii* % cover, total seagrass % cover, total SAV % cover, and total SAV canopy height. As in previous studies in the same region [24–26], S*yringodium filiforme* was rarely encountered (n = 10, 1.6% of total samples) and thus was not further considered. Ultimately, potential limiting variables were confined to the following four: temperature, salinity, water depth, and SAV % cover. Due to lack of SAV data for 2007, specific analyses that included SAV covered only the period from 2008 forward.

Multiple QR functional response shapes were investigated including linear, quadratic, cubic, log-linear, natural cubic splines (function ‘ns’ of package ‘splines’) [48], and additive quantile smoothing spline (AQSS) response curves (functions ‘rqss’ and ‘qss’ of package ‘quantreg’ [49,50]). Natural cubic splines were constructed with 3 (0.25, 0.50, and 0.75 quantiles of the predictor), 2 (0.33 and 0.66 quantiles of the predictor) and 1 (0.5 quantile of the predictor) internal knots [48]. QR coefficient confidence intervals were constructed and tested for significance by *xy*-pair bootstrapping (function ‘summary.rq’ of package ‘quantreg’). Natural cubic spline QRs were modeled without intercepts; these QRs were considered significant if each individual spline describing a sub-range of the data was significant.

#### Spatiotemporal relationships

Heatmaps were generated to visualize spatiotemporal trends in pink shrimp density and the habitat attributes found by QR to potentially limit pink shrimp density. Observation data were converted to 47 row by 20 column matrices to display their spatial (47 sampling sites) and temporal (10 year x 2 seasons = 20 year-seasons) patterns, and color gradients were used to represent the magnitude of density.

Procrustean analyses allowed direct testing of statistical concordance between matrices of shrimp density and habitat attributes [51–53]. Procrustean analysis minimizes the residual sum of squares between a target matrix (**X**: here, shrimp density) and a second, fitted matrix (**Y**: here, habitat attributes), superimposed on it by scaling, rotating, and dilating [51,52]. The Procrustean Sum of Squares (PSS, also known as Gower’s Statistic: m^2^_**X**,**Y**_) represents the minimized residual sum of squares from the fitting procedure and is used to assess Procrustean fit ranging from 0 to 1, with higher values presenting poorer fit [51–53]. The PSS metric is equivalent to 1 –*r*^2^, where *r* is a Pearson correlation coefficient [52]. Because the method hinges on one-to-one relationships between the matrices being compared, Procrustean analysis [54,55] cannot accommodate missing values. Following Adams et al. [54] and Arbour and Brown [55], missing habitat attribute values were imputed with linear regressions that included site, season, and year as potential factors. PROTEST (function ‘protest,’ package ‘vegan’, permutation n = 9999) provided statistical significance of Procrustean fits between density and habitat attribute matrices [51].

#### Pink shrimp density and habitat attributes among density clusters

Hierarchical clustering procedures were used to identify groups with similar density patterns among sites or year-seasons [56,57]. Bray-Curtis dissimilarity matrices were constructed (‘vegdist’ function, ‘vegan’ package) with respect to site (i.e., spatial) and year-season (i.e., temporal) density observations. Hierarchical agglomerative clustering (function ‘hclust’) using the “Ward.D2” agglomeration method identified spatially and temporally similar density groupings. *A prioiri* statistical significance of clusters was tested via similarity profiling (function ‘simprof’ of package ‘clustsig’) (permutations = 999, number of expected groups = 1000) of identified density cluster memberships [57]. Permutational multivariate ANOVA (PERMANOVA: function ‘adonis2’ of package ‘vegan’) testing provided *a posteriori* cluster significance [58,59]. PERMANOVA was also used to investigate inter-annual and seasonal density differences using year-season cluster membership as a categorical nesting factor. To investigate potential dispersion influences on PERMANOVA significance, multivariate homogeneity of dispersions analysis (function ‘betadisper’ of package ‘vegan’) was used to test for inter-cluster differences in dispersion (i.e., distance to centroid) [60]. The density heat map was rearranged to display site and year-season cluster membership.

Pink shrimp density and habitat attributes previously detected via QR as potentially limiting to pink shrimp density were investigated among site and year-season clusters. First, medians and confidence intervals (CI) of density and habitat attributes were computed for each site cluster and year-season cluster. Confidence intervals (CIs) about median values were computed as:

1.58*IQR/sqrt(n) (1)

where IQR = interquantile ranges and n = sample size, as described in McGill et al. [61] and Chambers et al. [62]. Plots of density and habitat attributes’ median, CIs, minimum, and maximum values were used to visualize their distributions within site and year-season clusters. Density and habitat attributes were analyzed with respect to site or year-season clusters. Nonparametric tests were used because parametric normality and equality of variance assumptions were usually violated. Kruskal-Wallis tests were used to investigate differences in distribution shape and range (i.e., location: [63]) of density and habitat attributes among site or year-season clusters. Post-hoc Tukey-type nonparametric Conover multiple comparison tests (function ‘posthoc.tukey.conover.test’ of package ‘PMCMR’) were used to test for significant pairwise differences. These tests were implemented as χ^2^ distributions to correct for data ties, and p-values were Bonferroni-corrected [63,64]. A series of correlation analyses was used to identify habitat attribute distribution characteristics that associated with site or year-season cluster median densities. Pearson correlation analyses were applied to median, minimum, maximum, and standard deviation of habitat attributes within site or year-season clusters.

## Results

A total of 3,178 farfantepenaeid shrimp specimens were collected. The distribution of shrimp sizes suggested a gear capture inefficiency for individuals <5mm CL; therefore, data only for shrimps ≥5 mm CL (2,417 shrimps) were retained for further analysis (S1 Fig). Of the retained shrimp, 1,573 (65.1%) were identified as pink shrimp, *F*. *duorarum*, and the remaining 844 (34.9%) were identified as farfantepenaeids due to difficulties with species identification of individuals <8 mm CL. Of the 1,937 individuals with measured CL, 1,931 individuals (79.9%) were considered juveniles (≤17.5 mm CL) and the remaining 36 individuals as subadults.

Pink shrimp density observations ranged from 0 to 13.0 shrimp m^-2^; 105 instances (11.2%, N = 940 samples) of densities ≥2 shrimp m^-2^ were observed, while no farfantepenaeid shrimps were observed in 377 samples (40.1%). Overall, shrimp density averaged 0.86 (SD = 1.32) shrimp m^-2^ and was significantly lower (t_(α = 0.10,2),939_ = -26.53, P <0.0001) than the 2 shrimp m^-2^ CERP Interim Goal threshold. Average density in any year-season was always < 2.0 shrimp m^-2^ (Table 1), although the highest year-season density (2014 Dry: 1.62 ± 2.02 shrimp m^-2^; Table 1) did not significantly differ from 2 shrimp m^-2^ (t_(α = 0.10,2),46_ = -1.30, p > 0.10). Averaged over all sites, mean dry season shrimp densities were higher than those of the subsequent wet season 50% of the time. Averaged over year-seasons, the highest mean site density was 2.15 (±1.95) shrimp m^-2^ at site 33 (Table 2). Seven sites (7, 10, 12, 33, 34, 43, and 44: 14.9%) exhibited temporally averaged densities that did not significantly differ from 2.0 shrimp m^-2^ (t_(α = 0.10,2),19_ = -0.080, -1.69, -0.30, 0.34, -1.63, -1.57, -0.32, respectively; p > 0.10). Most sites (n = 33, 70.2%) exhibited average densities below 1 shrimp m^-2^ (Table 2).

**Table 1:** Number of pink shrimp collected, average pink shrimp density (± SD), and average (± SD) of water quality and habitat attributes for survey year-seasons.

Year	Season	# Shrimp	Density (# m^-2^)	Temp (°C)	Sal (ppt)	Depth (m)	SAV (% Cover)
2007	Dry	131	0.93 ± 1.11	23.90 ± 1.99	26.11 ± 5.62	0.67 ± 0.18	ND
	Wet	63	0.45 ± 0.9	30.81 ± 1.07	20.36 ± 5.98	0.72 ± 0.17	ND
2008	Dry	165	1.17 ± 1.95	22.29 ± 1.44	25.28 ± 4.12	0.73 ± 0.17	52.45 ± 26.52
	Wet	104	0.74 ± 1.09	29.84 ± 0.99	23.57 ± 5.32	0.73 ± 0.17	70.38 ± 15.97
2009	Dry	198	1.4 ± 1.4	21.61 ± 1.53	25.95 ± 5.61	0.63 ± 0.15	78.36 ± 14.13
	Wet	56	0.4 ± 0.58	31.25 ± 1.85	22.93 ± 6.47	0.65 ± 0.15	75.25 ± 20.76
2010	Dry	98	0.7 ± 0.94	19.28 ± 2.89	25.50 ± 2.90	0.59 ± 0.14	62.28 ± 23.08
	Wet	123	0.87 ± 1.34	31.62 ± 1.17	24.18 ± 6.92	0.71 ± 0.20	63.46 ± 31.63
2011	Dry	98	0.7 ± 0.9	21.31 ± 1.83	27.09 ± 3.02	0.60 ± 0.18	72.35 ± 19.22
	Wet	112	0.79 ± 0.99	31.72 ± 1.56	31.86 ± 3.68	0.73 ± 0.22	69.96 ± 22.34
2012	Dry	182	1.29 ± 1.73	22.52 ± 1.00	24.47 ± 3.45	0.71 ± 0.16	83.74 ± 12.07
	Wet	182	1.29 ± 1.65	31.10 ± 1.85	15.30 ± 5.64	0.73 ± 0.15	65.44 ± 16.61
2013	Dry	82	0.58 ± 0.68	21.45 ± 1.07	28.85 ± 3.41	0.86 ± 0.18	57.98 ± 19.80
	Wet	29	0.21 ± 0.43	29.30 ± 0.87	15.98 ± 7.24	0.79 ± 0.18	60.07 ± 17.74
2014	Dry	228	1.62 ± 2.02	22.89 ± 1.39	23.33 ± 5.71	0.69 ± 0.15	61.41 ± 20.49
	Wet	15	0.11 ± 0.22	29.44 ± 1.12	29.30 ± 4.84	1.02 ± 0.20	53.20 ± 20.96
2015	Dry	92	0.65 ± 0.81	26.06 ± 1.48	28.85 ± 3.42	0.74 ± 0.19	68.51 ± 19.71
	Wet	204	1.45 ± 2.25	30.87 ± 1.45	23.11 ± 6.19	0.80 ± 0.17	64.23 ± 19.75
2016	Dry	119	0.84 ± 1.18	26.03 ± 2.06	18.60 ± 6.08	0.77 ± 0.20	60.63 ± 21.48
	Wet	136	0.96 ± 0.93	30.28 ± 1.22	12.22 ± 2.94	0.83 ± 0.17	59.73 ± 21.19
Overall	Dry	1393	0.99 ± 1.38	22.73 ± 2.65	25.40 ± 5.27	0.70 ± 0.19	66.54 ± 22.00
	Wet	1024	0.73± 1.25	30.65 ± 1.53	21.87 ± 8.10	0.77 ± 0.20	64.64 ± 21.93

ND = No Data

**Table 2:** Number of pink shrimp collected, average pink shrimp density (± SD), and average (± SD) of water quality and habitat attributes for survey sites.

Site	# Shrimp	Density (# m^-2^)	Temp (°C)	Sal (ppt)	Depth (m)	SAV (% Cover)
1	73	1.22 ± 1.65	27.42 ± 4.04	29.03 ± 5.09	0.84 ± 0.14	71.44 ± 20.10
2	57	0.95 ± 1.54	26.90 ± 4.23	26.75 ± 4.92	0.88 ± 0.17	70.37 ± 19.51
4	35	0.58 ± 1.49	26.68 ± 4.06	27.78 ± 4.94	0.79 ± 0.2	74.40 ± 16.07
5	66	1.10 ± 1.93	26.78 ± 4.06	25.67 ± 5.09	0.76 ± 0.19	67.94 ± 21.58
6	19	0.32 ± 0.69	26.58 ± 3.89	25.10 ± 5.55	0.65 ± 0.14	79.31 ± 14.20
7	117	1.95 ± 2.80	26.72 ± 4.20	27.05 ± 5.10	0.63 ± 0.15	72.07 ± 15.29
8	27	0.45 ± 0.64	26.68 ± 4.23	26.05 ± 5.63	0.5 ± 0.12	67.45 ± 18.53
9	85	1.42 ± 1.10	26.58 ± 4.00	25.23 ± 5.82	0.59 ± 0.15	51.46 ± 19.84
10	94	1.57 ± 1.15	26.14 ± 3.87	24.97 ± 5.91	0.73 ± 0.18	77.71 ± 18.04
11	80	1.33 ± 1.36	26.31 ± 3.65	24.91 ± 5.99	0.66 ± 0.15	68.94 ± 17.80
12	111	1.85 ± 2.27	26.34 ± 3.64	24.46 ± 6.42	0.86 ± 0.17	73.90 ± 19.70
13	51	0.85 ± 1.11	26.41 ± 3.77	24.53 ± 6.17	0.8 ± 0.15	80.85 ± 10.49
14	45	0.75 ± 1.65	26.70 ± 3.75	23.75 ± 6.17	0.8 ± 0.15	82.69 ± 14.27
15	13	0.22 ± 0.36	26.36 ± 4.10	23.10 ± 5.46	0.75 ± 0.18	71.29 ± 18.34
16	36	0.60 ± 0.88	26.67 ± 3.96	22.78 ± 5.20	0.77 ± 0.21	72.08 ± 22.62
17	57	0.95 ± 1.77	26.58 ± 4.00	23.07 ± 5.26	0.86 ± 0.15	75.96 ± 17.11
18	9	0.15 ± 0.23	26.75 ± 4.13	17.10 ± 9.03	0.64 ± 0.17	70.33 ± 17.95
19	36	0.60 ± 0.65	26.77 ± 4.05	17.71 ± 7.82	0.7 ± 0.17	66.30 ± 21.90
20	38	0.63 ± 0.71	26.76 ± 3.99	17.72 ± 7.59	0.73 ± 0.22	54.80 ± 22.54
21	29	0.48 ± 0.64	26.68 ± 4.37	18.35 ± 6.88	0.68 ± 0.22	59.85 ± 26.64
22	33	0.55 ± 0.60	26.82 ± 4.64	18.93 ± 6.82	0.7 ± 0.2	57.64 ± 23.73
23	30	0.50 ± 0.72	26.84 ± 4.77	17.98 ± 7.25	0.67 ± 0.2	62.61 ± 19.56
24	12	0.20 ± 0.23	27.25 ± 5.18	18.29 ± 7.34	0.64 ± 0.19	66.70 ± 17.74
25	12	0.20 ± 0.35	27.05 ± 4.83	18.20 ± 6.45	0.65 ± 0.15	54.74 ± 23.68
26	27	0.45 ± 0.74	27.08 ± 5.07	20.53 ± 5.51	0.7 ± 0.14	53.51 ± 20.39
27	41	0.68 ± 0.96	26.73 ± 4.74	20.60 ± 5.79	0.67 ± 0.17	66.51 ± 19.81
28	29	0.48 ± 0.51	26.82 ± 4.38	20.51 ± 5.86	0.85 ± 0.19	38.07 ± 21.13
29	49	0.82 ± 1.02	26.82 ± 4.43	20.69 ± 5.78	0.79 ± 0.2	49.53 ± 23.86
30	33	0.55 ± 0.55	26.76 ± 4.09	22.13 ± 5.32	0.74 ± 0.23	53.08 ± 19.99
31	38	0.63 ± 0.69	27.27 ± 4.59	21.92 ± 6.26	0.77 ± 0.19	55.20 ± 23.45
32	43	0.72 ± 0.78	27.49 ± 4.91	22.06 ± 5.89	0.78 ± 0.23	55.56 ± 18.24
33	129	2.15 ± 1.95	26.80 ± 4.61	22.00 ± 6.02	0.82 ± 0.2	64.78 ± 15.60
34	88	1.47 ± 1.46	26.79 ± 4.79	22.50 ± 5.80	0.76 ± 0.22	63.24 ± 18.32
35	52	0.87 ± 0.84	26.90 ± 4.79	22.43 ± 6.96	0.74 ± 0.22	56.12 ± 23.22
36	52	0.87 ± 0.98	26.64 ± 5.11	22.04 ± 6.57	0.73 ± 0.18	53.76 ± 21.81
37	52	0.87 ± 1.13	26.43 ± 4.97	22.18 ± 6.88	0.81 ± 0.2	54.10 ± 22.31
38	33	0.55 ± 0.60	27.00 ± 5.28	21.01 ± 7.61	0.77 ± 0.18	51.91 ± 22.86
39	63	1.05 ± 2.89	27.24 ± 5.93	24.51 ± 6.65	0.84 ± 0.17	41.68 ± 22.78
40	76	1.27 ± 1.28	26.48 ± 5.57	27.16 ± 5.94	0.82 ± 0.17	79.73 ± 12.79
41	74	1.23 ± 1.24	26.31 ± 5.42	27.53 ± 5.76	0.69 ± 0.17	73.72 ± 18.74
42	42	0.70 ± 1.27	26.51 ± 5.34	28.47 ± 5.85	0.85 ± 0.22	66.68 ± 21.59
43	92	1.53 ± 1.33	26.17 ± 5.17	28.51 ± 6.09	0.68 ± 0.17	72.63 ± 17.57
44	113	1.88 ± 1.65	25.98 ± 5.06	28.77 ± 6.18	0.54 ± 0.16	59.35 ± 15.19
45	12	0.20 ± 0.31	26.15 ± 5.71	29.83 ± 5.78	0.72 ± 0.17	89.86 ± 4.83
46	45	0.75 ± 1.00	25.87 ± 5.74	29.57 ± 6.03	0.58 ± 0.19	63.17 ± 17.5
47	10	0.17 ± 0.35	25.82 ± 5.69	30.02 ± 5.87	0.81 ± 0.18	89.86 ± 6.12
Overall	2417	0.86 ± 1.32	26.68 ± 4.52	23.64 ± 7.05	0.74 ± 0.20	65.57 ± 21.97

Temperatures ranged from 12.49 to 36.06°C. Average temperatures demonstrated a clear pattern of cooler (22.73 ± 2.65°C) and warmer (30.65 ± 1.53°C) values for dry and wet seasons, respectively (Table 1). The dry season record was punctuated by an extreme cold front event that occurred during the 2010 field sampling. No pattern of variation in average temperatures among sites was readily discernable (Table 2). Salinities ranged from 2.48 to 39.71 ppt; overall average salinity was 23.64 (± 7.05) ppt (Table 2). Spatially averaged wet season salinities were generally lower than those of dry seasons, although 2011, 2014, and 2015 wet seasons were notable exceptions with higher average salinity than both the preceding and following dry seasons (Table 1). Sampling sites’ mean salinity and standard deviation of salinity were negatively correlated (Pearson r = -0.63, t = -5.49, d.f. = 45, p < 0.0001; S2 Fig). Prior to epifaunal sampling during the 2011 and 2015 wet seasons, hypersaline (>40 ppt) conditions of considerable duration were recorded by continuous salinity loggers deployed within this sampling domain [65]. Only four temporally averaged site salinities were mesohaline, most (n = 42) were polyhaline, and one was euhaline (Table 2). Water depths ranged from 0.19 to 1.5 m and averaged 0.74 (± 0.20) overall (Table 2) with no appreciable trends among year-seasons or among sites. Total SAV % cover ranged from 4.57 to 100% and averaged 66.57% (± 21.97) with no clear year-season variation patterns (Tablea 1, 2). A planktonic microalgal bloom event was observed in parts of the Biscayne Bay coastal area during the 2013 wet season [65,66].

### Habitat limitations on pink shrimp density

**Fig 2:**
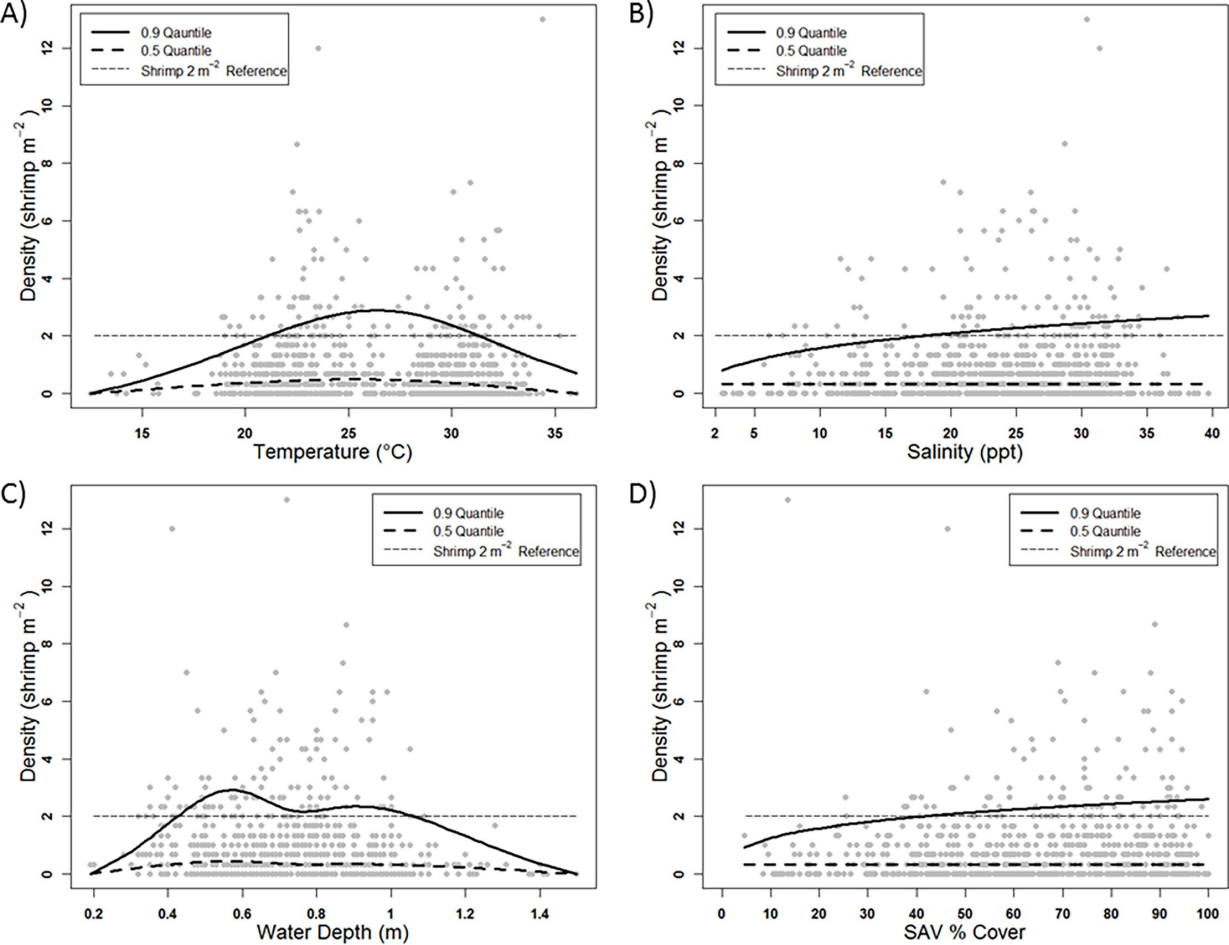
Pink shrimp density (shrimp m^-2^) and back-transformed 0.50 and 0.90 quantile regressions lines of predicted density (LN x+1) plotted against A) temperature (°C), B) salinity (ppt), C) water depth (m), and D) submerged aquatic vegetation (SAV: % cover). Predicted regression lines depict relationships reported in Table 2.

Of the multiple habitat attributes investigated, significant QR analysis results revealed that temperature (°C), salinity (ppt), water depth (m), and SAV (% cover) potentially limited pink shrimp density (Table 3, Fig 2). QR of density vs. temperature yielded a single-knot natural cubic spline relationship which was roughly dome-shaped and maximized at 26.6°C, with tails that tapered off at higher and lower temperatures (Table 3, Fig 2A). Temperatures between 21.08 and 31.33°C did not appear to limit pink shrimp densities to <2 shrimp m^-2^ (Table 3, Fig 2A). Although a series of functional shapes was considered for the QR density vs. salinity response curve, only the linear and log-linear responses were both significant and ecologically plausible [67]. The log-linear response, which suggested more severe density limitation below10 ppt and appeared more asymptotic at salinities above 10 ppt (Table 3, Fig 2B), seemed more plausible than the linear response. Salinities < ~18 ppt limited shrimp density to <2 shrimp m^-2^ (Table 3, Fig 2B). QR of pink shrimp density against water depth (m) yielded a 3 knot (0.25, 0.5, 0.75 quantile) splined bimodal relationship with steep increases in limitation below ~0.6 m and above ~ 1.0 m (Table 3, Fig 2C). Apparent constraint of density to <2 shrimp m^-2^ occurred at water depths less than 0.43 m and greater than 1.05 m (Table 3, Fig 2C). Shrimp density had a logarithmic linear relationship with SAV % cover (Table 3, Fig 2D). SAV cover less than 45% limited density to <2 shrimp m^-2^ (Table 3, Fig 2D). For the four habitat attributes, significant QRs were observed at the 0.9 quantile, but not at the 0.5 quantile (Table 3).

**Table 3:** Statistical significance of 0.5 and 0.9 quantile regressions of pink shrimp density (shrimp m^-2^: LN([x+1]) against temperature (°C), salinity (ppt), water depth (m), and submerged aquatic vegetation (SAV: % cover).

Quantile	Predictors	Coefficients (± SE)	t value	p value
0.5	Spline1(Temperature)	-0.59 ± 0.057	10.39	0.5551
	Spline2(Temperature)	-0.33 ± 0.16	-2.08	0.0375
0.9	Spline1(Temperature)	2.24 ± 0.094	23.80	< 0.0001
	Spline2(Temperature)	-0.49 ± 0.22	-2.26	0.0242
0.5	LN(Salinity)	0.00 ± 0.044	0.00	1.0000
	Intercept	0.29 ± 0.12	2.44	0.0147
0.9	LN(Salinity)	0.26 ± 0.091	3.17	0.0016
	Intercept	0.34 ± 0.28	1.22	0.2213
0.5	Spline1(Water Depth)	0.27 ± 0.053	5.04	< 0.0001
	Spline2(Water Depth)	0.15 ± 0.068	2.26	0.0243
	Spline3(Water Depth)	0.57 ± 0.14	4.07	0.0005
	Spline4(Water Depth)	-0.22 ± 0.16	-1.43	0.1544
0.9	Spline1(Water Depth)	1.07 ± 0.11	10.04	< 0.0001
	Spline2(Water Depth)	0.75 ± 0.23	3.29	0.0011
	Spline3(Water Depth)	2.15 ± 0.18	11.70	< 0.0001
	Spline4(Water Depth)	-0.83 ± 0.32	-2.62	0.0089
0.5	LN(SAV)	0.00 ± 0.036	0.00	1.0000
	Intercept	0.29 ± 0.13	2.18	0.0298
0.9	LN(SAV)	0.21 ± 0.075	2.75	0.0061
	Intercept	0.33 ± 0.30	1.10	0.2733

LN = natural logarithm.

### Spatiotemporal relationships

**Fig 3:**
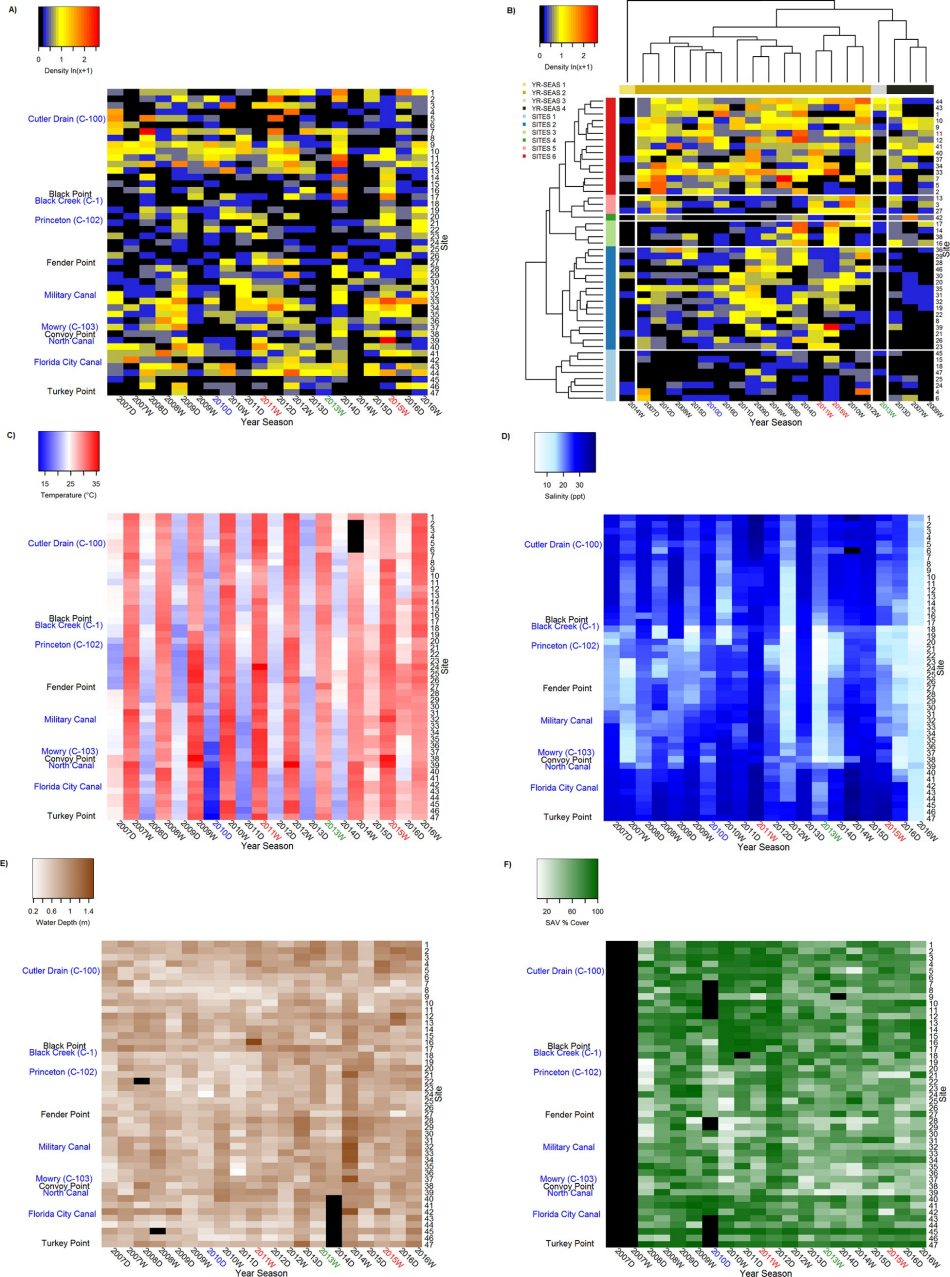
Heatmaps depicting spatial (i.e., site) and temporal (i.e., year-season) patterns in A and B) pink shrimp density (shrimp m^-2^: LN[x+1]), C) temperature (°C), D) salinity (ppt), E) depth (m), and F) SAV (% cover). Shrimp density patterns in panel B are organized by site and year-season cluster membership, as denoted by color bars along the left and top margins and presented in the legend. Black cells in A) and B) highlight 0 shrimp m^-2^ observations while in C) through F) black bars represent missing values. Year-season label colors depict ecological perturbations: red = hypersalinity event, blue = cold snap, green = algal bloom. Labels on the left margin of (A) refer to canal outlets (blue) and coastline features (black) depicted in Fig 1.

Heatmap visualization of pink shrimp spatiotemporal density trends revealed a general absence of pink shrimp from sites 13 to 28 (approximately Black Point to Fender Point, Fig 1) and sites 45 to 47 (near Turkey Point, Fig 1) across all year-seasons (Fig 3A). Within these groups of sites, only 16 (4.4%, N = 360) and 4 (6.7%, N = 60) instances of pink shrimp densities >2 shrimp m^-2^ were observed, respectively. Generally, higher densities were observed at sites 31 through 44 and sites 1 to 12, where 42 (15%, N = 280) and 40 (16.7%, N = 240) instances, respectively, of densities >2 shrimp m^-2^ were observed across all year-seasons. Densities were particularly low during the 2007, 2009, 2013, and 2014 wet seasons (< 0.5 shrimp m^-2^: Table 1), when shrimp were absent from a high proportion of samples (55.3, 51.1, 70.2, and 76.6%, respectively: Fig 3A). Other year-seasons (2008 dry, 2009 dry, 2012 dry, 2012 wet, 2014 dry, and 2015 wet: Fig 3A) exhibited relatively high average density (> 1 shrimp m^-2^), because the low and zero-catch observations from Black Point to Fender Point (Fig 1) were offset by higher density observations at most other sites (Fig 3A). Average densities in these six year-seasons yielded the highest year-season average densities, all >1 shrimp m^-2^ (Table 1).

Cluster analyses were performed to organize year-season elements and site-specific elements into groups according to shrimp density. Then, the heatmap of shrimp density (Fig 3A) was rearranged to reflect both the site and year-season cluster groupings (Fig 3B). The cluster groupings will be discussed in the following section. Heatmaps were developed to visualize spatiotemporal trends in temperature, salinity, water depth, and SAV (Fig 3C, 3D, 3E and 3F). Procrustean analyses revealed significant concordance of the shrimp density matrix (Fig 3A) with water depth, temperature, and salinity habitat attribute matrices (Fig 3C, 3D and 3E; Table 4) but not with the SAV matrix (Fig 3F, Table 4). Water depth and temperature exhibited the highest correlations, followed by salinity (Table 4). Each comparison yielded a high residual sum of squares (high m^2^_**X**,**Y**_ values), indicating relatively weak explanatory power of individual habitat attributes (Table 4). Procrustean fitting procedures explained 28.3, 27.1, and 22.1% of the variability in density for water depth, temperature, and salinity, respectively.

**Table 4:** Results of Procrustean analysis of density (shrimp m^-2^: LN([x+1]) relative to temperature (°C), salinity (ppt), water depth (m), and SAV (% cover) including goodness-of-fit measure (sum of squares, m^2^), correlation of the Procrustean rotation (r), and p value of the fit.

	m^2^	r	p value
Temperature	0.7287	0.5209	<0.0001
Salinity	0.779	0.4701	<0.0001
Water Depth	0.7169	0.5321	<0.0001
SAV	0.7777	0.4715	0.1162

### Pink shrimp density and habitat attributes among density clusters

SIMPROF testing identified six significant site clusters and four significant year-season clusters (Fig 3B). PERMANOVA testing of cluster membership confirmed SIMPROF site (F_5,41_ = 4.765, p = 0.001, R^2^ = 0.368) and year-season (F_7316_ = 3.727, p = 0.001, R^2^ = 0.411) clustering. Two site clusters (i.e., 2 and 6: Fig 3B) together included most (66%, n = 31) of the sampling sites. One large year-season cluster included most year-seasons (75%, n = 15: Fig 3B). Smaller year-season clusters (80%, n = 4) were mostly comprised of wet seasons having pink shrimp densities <0.5 shrimp m^-2^. Substantial differences in shrimp densities of members of different clusters likely drove the significantly differing multivariate dispersion among site (F_5,41_ = 14.886, p < 0.0001) and year-season (F_3,16_ = 22.987, p = <0.0001) clusters. PERMANOVA testing detected significant season (F_1,9_ = 1.912, p = 0.0063), but not year (F_9,9_ = 1.020, p = 0.4279), categorical temporal effects. Multivariate dispersions differed greatly among years (F_9,10_ = 5.56*10^29^, p < 0.0001). Significant seasonal multivariate dispersion (F_1,18_ = 7.047, p = 0.0161) was also observed with greater variability in the wet season than in the dry season.

**Fig 4:**
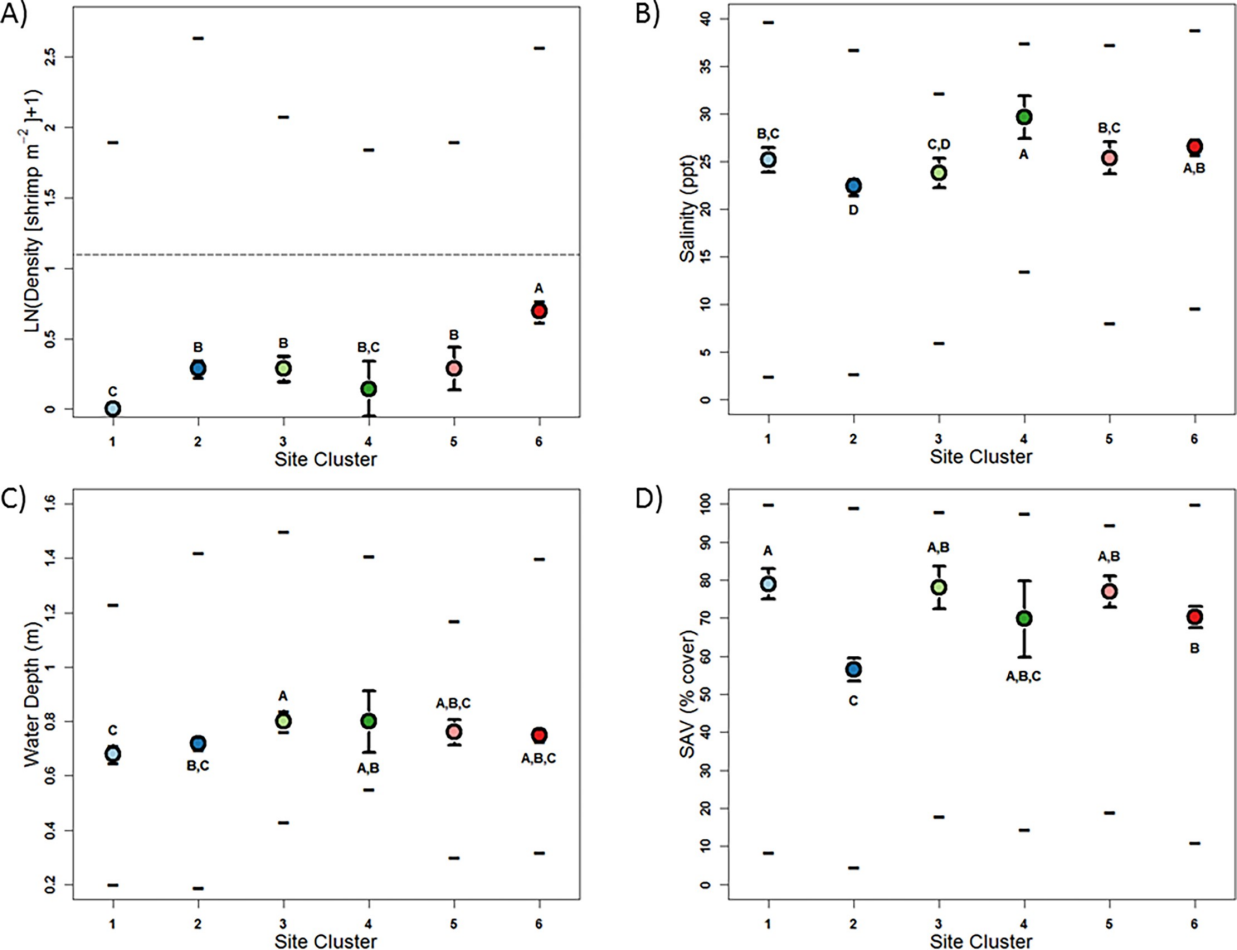
Median (± CI) and maximum, and minimum values of A) density (shrimp m^-2^: LN([x+1]), B) salinity (ppt), C) water depth (m), and D) submerged aquatic vegetation (SAV: % cover) in shrimp density site clusters. Point colors coincide with Figs 1 and 3B. Letters denote statistically similar groups. Horizontal line of A) depicts the 2-shrimp m^-2^ CERP Interim Goal.

**Fig 5:**
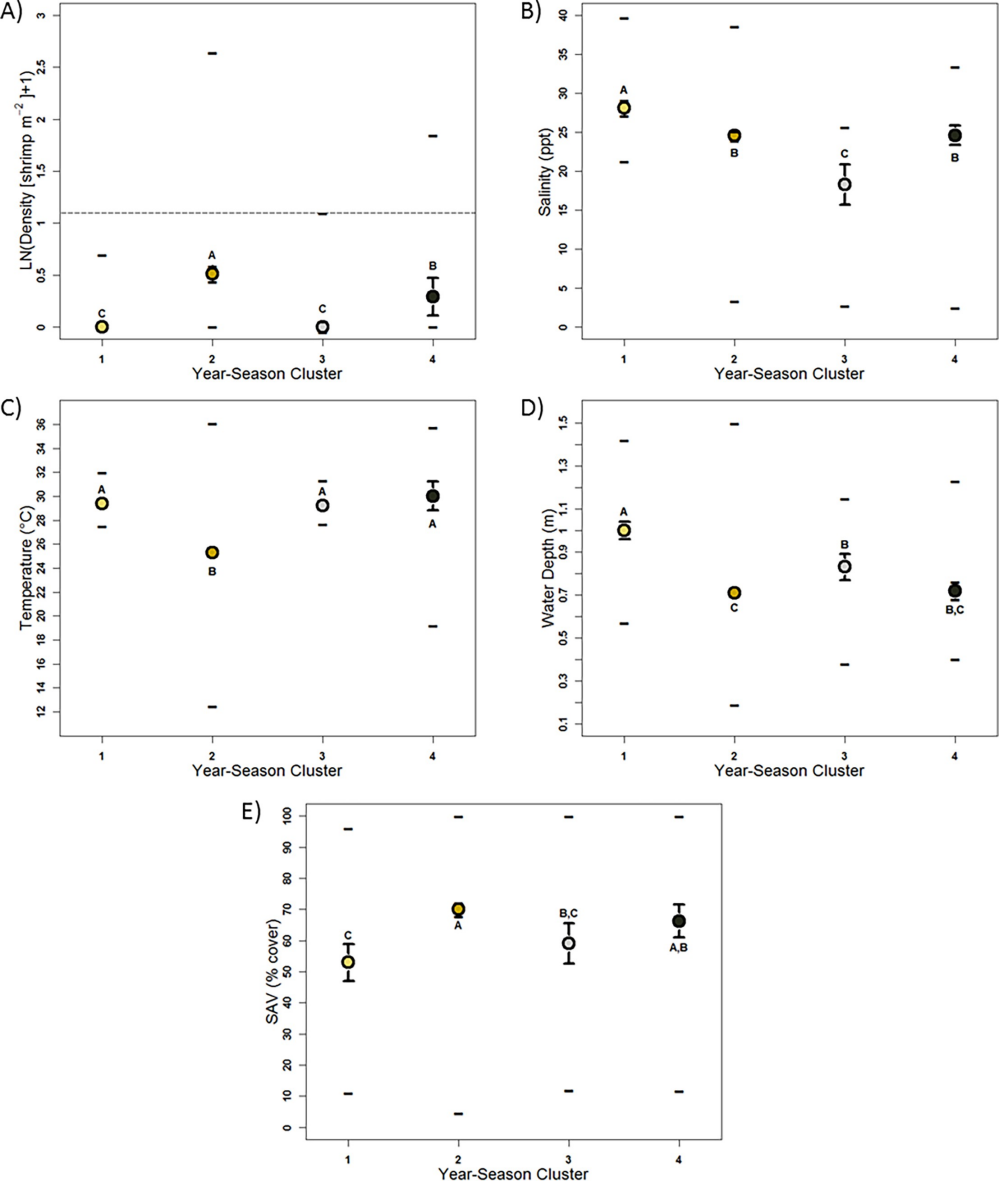
Median (± CI) and maximum, and minimum values of A) density (shrimp m^-2^: LN([x+1]), B) temperature (°C), C) salinity (ppt), D) water depth (m), and E) submerged aquatic vegetation (SAV: % cover) relative to shrimp density year-season clusters. Point colors coincide with Fig 3B. Letters denote statistically similar groups.

Significant differences in shrimp density distributions were detected among both site and year-season clusters (Table 5, Fig 4A, Fig 5A). Site clusters represented three relative median density levels: high (~0.7 shrimp m^-2^: site cluster 6); intermediate (~0.3 shrimp m^-2^: site clusters 2, 3, and 5); and low (0.0 and ~0.14 shrimp m^-2^: site clusters 1 and 4, respectively: Table 5, Fig 4A). Year-season clusters also exhibited three relative density levels: high (0.51 shrimp m^-2^: year-season cluster 2), intermediate (0.29 shrimp m^-2^: year-season cluster 4) and low density (0.0 shrimp m^-2^: year-season clusters 1 and 3: Table 6, Fig 5A). Significant differences among site clusters defined by differences in shrimp density were also observed for salinity, water depth, and SAV distributions (Table 5; Fig 4B, 4C and 4D), but temperature distributions did not differ among site clusters. All four habitat attributes exhibited significant differences among year-season clusters (Table 6, Fig 5B, 5C, 5D and 5E).

**Table 5:** Median and ~95% CI of density (shrimp m^-2^: LN([x+1]), temperature (°C), salinity (ppt), water depth (m), and submerged aquatic vegetation (SAV: % cover) and the χ^2^, d.f., and p values associated with Kruskal-Wallis testing of density clusters relative to site.

Site Cluster	n	Density	Temperature	Salinity	Water Depth	SAV
1	160	0.00 ± 0.036	27.73 ± 1.04	21.15 ± 1.29	0.68 ± 0.032	79.00 ± 4.00
2	320	0.29 ± 0.061	28.50 ± 0.71	22.38 ± 0.87	0.72 ± 0.024	56.50 ± 2.97
3	80	0.29 ± 0.090	28.05 ± 1.32	23.79 ± 1.54	0.80 ± 0.039	78.00 ± 5.65
4	20	0.14 ± 0.20	28.70 ± 3.32	29.67 ± 2.24	0.80 ± 0.113	69.75 ± 10.05
5	60	0.29 ± 0.15	28.60 ± 1.58	25.37 ± 1.69	0.76 ± 0.047	77.00 ± 4.13
6	300	0.69 ± 0.074	27.80 ± 0.72	26.57 ± 0.79	0.75 ± 0.025	70.25 ± 2.86
χ^2^	NA	148.27	0.64	71.07	21.41	98.24
d.f.	NA	5	5	5	5	5
p value	NA	<0.0001	0.9861	<0.0001	0.0007	<0.0001

Median CI computed as described in the text. Values are depicted in Fig 4.

**Table 6:** Median and ~95% CI of density (shrimp m^-2^: LN([x+1]), temperature (°C), salinity (ppt), water depth (m), and submerged aquatic vegetation (SAV: % cover) and the χ^2^, d.f., and p values associated with Kruskal-Wallis testing of density clusters relative to year-season.

Year-Season Cluster	n	Density	Temperature	Salinity	Water Depth	SAV
1	47	0.00 ± 0.00	29.40 ± 0.25	28.12 ± 0.96	1.00 ± 0.041	53.00 ± 5.94
2	705	0.51 ± 0.075	25.30 ± 0.48	24.55 ± 0.59	0.71 ± 0.015	70.00 ± 2.10
3	47	0.00 ± 0.051	29.20 ± 0.28	18.26 ± 2.59	0.83 ± 0.60	59.00 ± 6.51
4	141	0.29 ± 0.18	30.01 ± 1.20	24.58 ± 1.25	0.72 ± 0.40	66.25 ± 5.36
χ^2^	NA	89.77	32.28	70.07	85.68	22.03
d.f.	NA	3	3	3	3	3
p value	NA	<0.0001	<0.0001	<0.0001	<0.0001	<0.0001

Median CI computed as described in the text. Values in depicted in Fig 5.

## Discussion

Analysis of 10 years of monitoring data revealed few instances (11.2%) of pink shrimp densities > 2 shrimp m^-2^, the IG for focal Biscayne Bay pink shrimp populations [1]. All but one spatially averaged year-season density and all but a few temporally averaged site densities in the 2007–2016 database were significantly below 2 shrimp m^-2^. CERP implementation is expected to result in more favorable salinity conditions for pink shrimp, leading to higher shrimp densities [1,5,27]. Reductions in extreme salinity variability in the southern half of the study area (i.e., Black Point to Convoy Point: Fig 1) could lead to average densities > 2 shrimp m^-2^ across the entire study spatial domain. However, our results suggest that ~10ppt salinity (i.e., low mesohaline to oligohaline: Fig 2B) was a threshold below which pink shrimp densities were severely limited. Above this threshold, the positive response to salinity continued more gradually. The shape of the response may have been affected by the lack of pink shrimp density observations at salinities >39.71 ppt in this study. Limitation of pink shrimp densities at salinities <10 ppt does not support CERP post-restoration IGs of >2 shrimp m^-2^ with reduction of Biscayne Bay nearshore salinity regimes to oligohaline and low mesohaline conditions. Pink shrimp densities reported here represent an underestimate of true density as a substantial number of shrimps (~25%) were removed from analysis due to concern about gear sampling inefficiency of shrimps <5 mm CL.

Spatial pink shrimp density patterns from Black Point to Convoy Point (Fig 1) were represented by membership in one low density site cluster (cluster 1) and one intermediate density site cluster (cluster 2). This zone is strongly influenced by canal discharges [11,23–26,33,68]. Both rapid (<60 min to 2 d) and extreme (~25 ppt) salinity reductions can occur along this stretch of coastline [22,24,25,29,69,70]. Such salinity fluctuations can alter fish community assemblages [23] and may affect foraging behavior and survival [22,23]. Pink shrimp may avoid these conditions as they have been reported to migrate to avoid large-volume riverine inflows [71]. Rapid salinity reductions of greater than 20 ppt cause near complete pink shrimp mortality in laboratory settings [72–74]. Low and intermediate density site clusters 1 and 2 included sites 45, 46, and 47 (Figs 3 and 4), which are located near Turkey Point (Fig 1), well south of the canal zone (Black Point to Convoy Point) and so were not impacted by low and variable salinity conditions. These sites had moderate minimum salinities (≥11.06 ppt), the highest temporally-averaged salinity across all sites (≥29.57 ppt: Table 2), and generally high SAV cover (Table 2, Fig 3F). The cause of their low shrimp density is unknown, but does not seem to be related to limitation due to salinity or SAV cover.

Site cluster 6, comprised of sites located away from canal mouths (Fig 1), had the highest median density and the second highest minimum salinity (9.62 ppt). Previous field [9,75] and modeling [35,76] studies describe the area represented by the more northern sites of this cluster as an area of relatively high shrimp abundance. These northern sites were situated immediately across the bay from a wide ocean inlet known as the Safety Valve. This inlet has been considered a primary postlarval immigration pathway [35,76] and may have contributed to high densities at northern sites [77,78]. Shrimp cumulative size frequency distributions differed between northern (sites 1–17) and southern (sites 18–47) sampling sites (S1 Fig). Comparison of these size distributions suggested that juvenile shrimps were more abundant in the north. This difference does not necessarily mean that greater recruitment was occurring in the north because differential growth and/or mortality also could have caused the size distribution difference. The inclusion of southerly sites (33, 34, 37, 40, 41, 43, and 44) within site cluster 6 (Figs 1 and 3B) contradicts the notion of southern recruitment limitation.

Some southern sites in high density cluster 6 are located near mangrove creeks that drain more natural watersheds. Other cluster 6 sites, both northern and southern, are located near, but not immediately adjacent to, canals with relatively small freshwater discharges (Military Canal: 1994–2003 annual mean canal output = 21.9 cfs; Cutler Drain C-100: 1994–2003 annual mean output = 46.1 cfs; [33]). However, sites immediately adjacent to the low-volume-discharge canals (i.e., 6 and 32) do not have high shrimp density and clustered with intermediate and low-density sites (clusters 1 and 2, respectively: Figs 1 and 3).

Most year-seasons (75%) were aggregated within one large cluster, indicating a general lack of inter-annual and inter-season variability in Biscayne Bay juvenile pink shrimp densities. However, year-season cluster 2, which had the highest median cluster shrimp density, consisted mainly (60%) of dry season sampling events. Smaller shrimp were associated with the higher dry season densities (S1 Fig), presumably because of higher dry season postlarval recruitment. Observation of higher densities in the dry rather than wet season is contrary to the pink shrimp IG, which focused on improving ‘peak’ fall (wet) season abundance [1]. The pink shrimp IG was based upon previous findings of a summer/fall (i.e., wet season) peak in abundance [8,9]. Others reported peak juvenile abundances in late fall/early winter (i.e., November/December) [12] or estimated maximal Biscayne Bay juvenile pink shrimp populations occurring in November (i.e., late fall) [75,79]. Differences in sampling gear and spatial domain and the short durations (≤2 yr) of the four pervious studies [8,9,75,79] complicate comparisons with the present study. Although of greater duration, the present study’s bi-annual sampling effort may have insufficient resolution to precisely identify the period of peak pink shrimp density or its year to year variation.

A lack of understanding of Biscayne Bay pink shrimp recruitment complicates study of their abundance patterns. The only study on recruitment reported a late fall through early winter (i.e., October through March: [80]) peak in recruitment, but was too short in duration (1 yr) to provide any information on inter-annual trends or consistency. This peak agreed with juvenile abundance studies reporting a late fall/early winter peak [75,79]. A model of pink shrimp postlarval recruitment from Tortugas and Marquesas spawning grounds found that oceanographic processes favored potential recruitment pathways up the Atlantic coast of the Florida Keys during late wet season and early dry season months [81]. Modeling of larval permit *Trachinotus falcatus* originating from spawning grounds near those of pink shrimp also found similar recruitment patterns for the Florida Keys and Biscayne Bay [82]. Oceanographic, coastal, and climatic conditions affect pink shrimp adult reproductive activity [83,84], and larval abundances [85] and interact with behavior to influence early life stage recruitment to nearshore areas [81,86–93]. Use of pink shrimp and other offshore spawning species as indicators of ecological conditions in their nearshore nursery grounds is complicated by life cycles affected by prior external conditions [5,32].

According to QR analysis, pink shrimp densities were significantly limited by four habitat attributes (water temperature, salinity, water depth, and SAV % cover). Two of these (i.e., salinity regime and SAV % cover) can be influenced by freshwater management within this study domain. Procrustean analysis confirmed the influence of each habitat attribute except SAV % cover. These habitat attributes vary at different time scales; for example, water depth can differ by as much as 1.3 m within 12 hr during extreme tidal cycles, whereas SAV % cover may integrate salinity, nutrients, water clarity, and other influential factors from 6 mo. to 1 yr or more. Responses on different time scales may affect the ability to discern relationships.

The correlation between spatiotemporal pink shrimp density and water depth was the strongest in this study. This was unexpected given the narrow spatial sampling domain along the mangrove-seagrass ecotone and the small range of variation in water depth in the data. Associations between nearshore pink shrimp abundance and depth have been previously reported [75,80,94,95]. Other studies that focused on very nearshore areas (<100 m from shore) also found higher abundances there [8–10,12,96]. Recruiting postlarval pink shrimp often concentrate in SAV near the low-tide mark along shorelines [9,12,97–102]. Other pink shrimp habitat investigations [80,94] found multiple habitat attributes (e.g., salinity, salinity standard deviation, standard deviation of turbidity, temperature, median sediment size, dissolved oxygen concentration, water depth, and benthic habitat characteristics) can influence pink shrimp abundance. As re-iterated by Zink et al. [6], Costello et al. [12] stated that “…factors other than salinity per se control abundance of the euryhaline juveniles…” The present results clearly indicate water depth is one of these factors.

Fluctuating tidal depths and/or reduced detection probability at greater depths likely contributed to the domed shape of the QR relationship [103]. Short-term variations in water depth are caused by astronomical tides and meterological/climatological events [104], which may override any effects of variation in freshwater inflow, especially in a relatively open bay area such as south-central Biscayne Bay. Hydrological alterations can both increase inundation [105] or decrease water depth via sediment accretion [106]. The multiple broad openings connecting Biscayne Bay to the open ocean promote dominance of water depth by astronomical tides [107,108]. Given the exposed nature of that part of Biscayne Bay’s coastline, changes in freshwater inflow within the plausible range that may occur along this coastline are unlikely to appreciably alter water depth within the domain. On longer time scales vertical movements of land surface and coastal geomorphology also can influence nearshore water depth and hydroperiod [104]. Sea level rise may also interact with hydrological changes to further alter coastal water levels and marsh inundation [109]. Although these processes are slow, sea level rise effects on water depth could influence nearshore pink shrimp density within the CERP time frame.

In this study, temperature emerged as the second most influential habitat attribute. The 90^th^ QR dome shaped response curve was shifted towards cooler water temperatures, which reflected the previously discussed higher densities observed during the dry (winter) season. As has already been discussed, seasonality, and thus temperature, relationships to density are dependent upon recruitment patterns. However, it is important to note here that pink shrimp are known to ‘overwinter’ in estuaries as far north as North Carolina [98] and can occur at higher abundance during winter periods at the southern limit of their range [110].

Along with temperature, salinity was indicated by Kinne [111,112] as a major factor influencing organismal abundance in estuaries. Re-analyses by Zink et al. [6] of data presented by Brusher and Ogren [113] and Minello [114] found increasing pink shrimp abundance with increasing salinity and no statistical difference between polyhaline and mesohaline conditions. It was unexpected to find no limitation of pink shrimp density at high salinities (>35 ppt), but a shortcoming of this study is lack of hypersaline observations (>40ppt). Perhaps the upper limit of salinity observations in our data (i.e., none >39.71 ppt) was not sufficient to reflect a reduction of densities that may occur under hypersaline conditions [5].

The Biscayne Bay pink shrimp IG proposed a pink shrimp preference for seagrasses and presumed that increased % cover of seagrasses would increase the seaward spatial extent of *H*. *wrightii* [30] and pink shrimp abundance [1,5,30]. Presently, total SAV QRs yielded the most plausible relationship between pink shrimp density and the benthic habitat metrics investigated. Procrustean analysis test results did not support this. The seemingly weak statistical relationships with either total or species-specific SAV metrics was unexpected. Pink shrimp associations with *H*. *wrightii* have been previously reported [12,96,97,115], while other studies have reported high pink shrimp densities associated with total SAV biomass or % cover [10,116,117]. Although one study reports negative impacts of drift and attached algal biomass [118], the positive relationships reported by most studies suggest a stronger relationship between pink shrimp density and either species-specific or total SAV than found in this study.

Several environmental perturbations occurred during the period covered by this study, but only one may have affected pink shrimp density. Variability in climatic conditions led to both wetter and drier than normal wet seasons (Fig 3D). However, departure from typical salinity regimes did not seem to influence temporal density patterns appreciably. For example, the second highest wet-season pink shrimp density (1.29 ± 1.65 shrimp m^-2^: Table 1) coincided with 2012 record rainfall that reduced salinities (3.34 to 22.08 ppt) across the spatial domain (Fig 3D). Conversely, the highest wet season pink shrimp average density (1.45 ± 2.25 shrimp m^-2^: Table 1) occurred during the 2015 wet season, previously denoted as a ‘hypersaline’ period [65]. Record dry season rainfall during 2016 yielded the lowest average dry season salinity (Table 1, Fig 3D) while pink shrimp densities that dry season were moderate (0.84 ± 1.18 shrimp m^-2^: Table 1). Despite their differing salinity conditions, these year-seasons were assigned to the same shrimp density cluster (Fig 3B). The 2013 wet season clustered separately from the others, suggesting a negative impact of microalgal bloom conditions [65,66] on pink shrimp density. No discernable impact on pink shrimp densities was observed after passage of an extreme cold front in the 2010 dry season.

Due to the field sampling design, the present study results may be applicable only to shallow areas < 100 m from the shoreline. Application of study results to areas further offshore should proceed with caution, if at all, due to potential interaction with other habitat attributes that influence trends in pink shrimp density. The present study was also limited by the apparent low catchability of very recently settled pink shrimp by the throw trap gear suggested by reduced numbers of pink shrimp from 3 to 5 mm CL (S1 Fig). Pink shrimp postlarvae are generally considered settled in their nursery habitat by 3 mm CL [9,12,86–88]. Pink shrimp postlarvae settle in shallow (≤1 m), calm water areas along shorelines [12,115], which would suggest they should be readily available to the present field sampling program that samples nearshore waters generally < 1 m deep.

The RECOVER Biscayne Bay IG has set >2 shrimp m^-2^ as a target wet season pink shrimp density to be achieved with CERP BBCW implementation. But achievement of BBCW and CERP salinity IGs, which include low mesohaline (<10 ppt) and even oligohaline conditions (<5 ppt), may not support increased pink shrimp density. The Biscayne Bay pink shrimp IG may need modification to clarify whether the ≥ 2 shrimp m^-2^ target refers to all monitoring observations or a seasonal or annual average density across the entire shoreline and to reconsider spatial and seasonal abundance patterns. The present 10 yr analysis of pink shrimp density patterns could be used to refine the RECOVER Biscayne Bay IG.

## Supporting information

S1 Fig Histograms depicting size frequencies (mm CL) of A) all farfantepenaeid shrimps collected, B) those collected north of Black Point, and C), those collected south of Black Point, D) those collected in the dry season, and E) those collected in the wet season. Vertical dashed line in A) separates smaller sizes (to left of line) that were removed from analysis due to suspected catchability concerns. Shrimp size frequency differences were detected between the two regions (D2-tailed = 0.230, p < 0.0001) and between the wet and dry seasons (D2-tailed = 0.092, p < 0.0001)
(TIF)

S2 Fig Salinity distributional trends of A) mean salinity (ppt) and B) standard deviation of salinity (ppt) across sampling sites across all year-seasons sampled while C) depicts the scatter of salinity mean and standard deviation values as well as the significant correlation trend line between them
(TIF)
