# Supplementary figures and images for: Pink shrimp Farfantepenaeus duorarum spatiotemporal abundance trends along an urban, subtropical shoreline slated for restoration
Source: PLoS One. 2018 Nov 7;13(11):e0198539. doi: 10.1371/journal.pone.0198539 (PMC6221258; doi:10.1371/journal.pone.0198539)

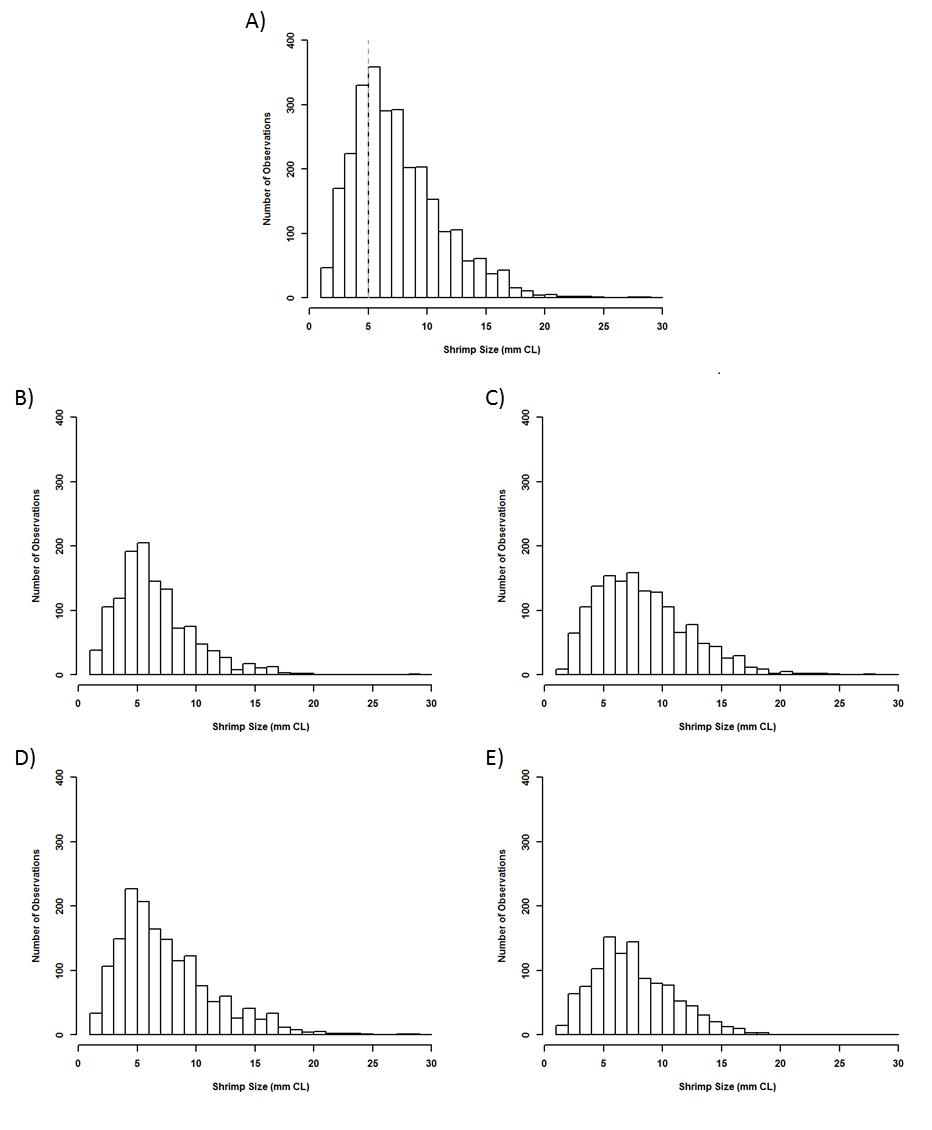

Supplement: S1 Fig — Histograms depicting size frequencies (mm CL) of A) all farfantepenaeid shrimps collected, B) those collected north of Black Point, and C), those collected south of Black Point, D) those collected in the dry season, and E) those collected in the wet season. Vertical dashed line in A) separates smaller sizes (to left of line) that were removed from analysis due to suspected catchability concerns. Shrimp size frequency differences were detected between the two regions (D2-tailed = 0.230, p < 0.0001) and between the wet and dry seasons (D2-tailed = 0.092, p < 0.0001) (TIF) [file pone.0198539.s001.tif]

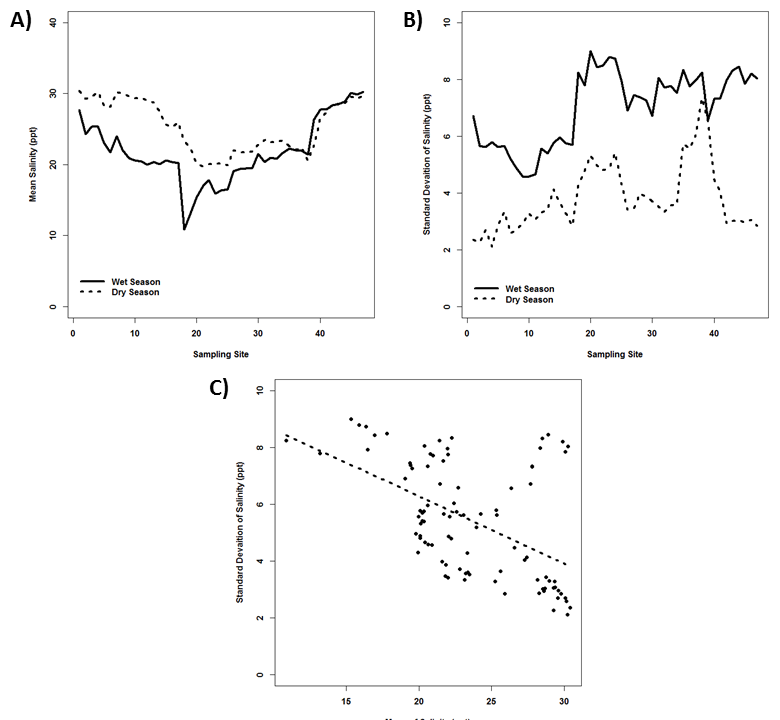

Supplement: S2 Fig — Salinity distributional trends of A) mean salinity (ppt) and B) standard deviation of salinity (ppt) across sampling sites across all year-seasons sampled while C) depicts the scatter of salinity mean and standard deviation values as well as the significant correlation trend line between them (TIF) [file pone.0198539.s002.tif]
